# Human Milk Oligosaccharide 2′-Fucosyllactose Modulates Local Viral Immune Defense by Supporting the Regulatory Functions of Intestinal Epithelial and Immune Cells

**DOI:** 10.3390/ijms231810958

**Published:** 2022-09-19

**Authors:** Veronica Ayechu-Muruzabal, Bente Poelmann, Alinda J. Berends, Nienke Kettelarij, Johan Garssen, Belinda van’t Land, Linette E. M. Willemsen

**Affiliations:** 1 Division of Pharmacology, Utrecht Institute for Pharmaceutical Sciences, Utrecht University, 3584 CG Utrecht, The Netherlands; 2 Danone Nutricia Research, 3584 CT Utrecht, The Netherlands; 3 Center for Translational Immunology, The Wilhelmina Children’s Hospital, University Medical Center Utrecht, 3584 EA Utrecht, The Netherlands

**Keywords:** viral infection, poly I:C, 2′-fucosyllactose, host-defense, moDC, T-cell

## Abstract

Human milk contains bioactive components that provide protection against viral infections in early life. In particular, intestinal epithelial cells (IEC) have key regulatory roles in the prevention of enteric viral infections. Here we established an in vitro model to study the modulation of host responses against enteric viruses mimicked by poly I:C (pIC). The effects of 2′-fucosyllactose (2′FL), abundantly present in human milk, were studied on IEC and/or innate immune cells, and the subsequent functional response of the adaptive immune cells. IEC were pre-incubated with 2′FL and stimulated with naked or Lyovec™-complexed pIC (LV-pIC). Additionally, monocyte-derived dendritic cells (moDC) alone or in co-culture with IEC were stimulated with LV-pIC. Then, conditioned-moDC were co-cultured with naïve CD4^+^ T helper (Th)-cells. IEC stimulation with naked or LV-pIC promoted pro-inflammatory IL-8, CCL20, GROα and CXCL10 cytokine secretion. However, only exposure to LV-pIC additionally induced IFNβ, IFNλ1 and CCL5 secretion. Pre-incubation with 2′FL further increased pIC induced CCL20 secretion and LV-pIC induced CXCL10 secretion. LV-pIC-exposed IEC/moDC and moDC cultures showed increased secretion of IL-8, GROα, IFNλ1 and CXCL10, and in the presence of 2′FL galectin-4 and -9 were increased. The LV-pIC-exposed moDC showed a more pronounced secretion of CCL20, CXCL10 and CCL5. The moDC from IEC/moDC cultures did not drive T-cell development in moDC/T-cell cultures, while moDC directly exposed to LV-pIC secreted Th1 driving IL-12p70 and promoted IFNγ secretion by Th-cells. Hereby, a novel intestinal model was established to study mucosal host-defense upon a viral trigger. IEC may support intestinal homeostasis, regulating local viral defense which may be modulated by 2′FL. These results provide insights regarding the protective capacity of human milk components in early life.

## 1. Introduction

Enteric viral infections are one of the most common infectious diseases in humans [1] among which rotavirus (RV) infections are recognized as the leading cause of severe dehydrating diarrhea and mortality in children [2]. RV is a double-stranded RNA virus (dsRNA) which typically infects epithelial cells of the small intestine thereby disrupting the enterocyte morphology and absorptive functions, leading to diarrhea in early life [3].

Intestinal epithelial cells (IEC) provide a physical barrier to protect the host from pathogens as well as playing an important role in immuno-surveillance [4]. Specialized IEC such as mucus-producing goblet cells, antimicrobial peptide secreting Paneth cells or enteroendocrine cells have developed strategies to support the protective function of IEC [5,6,7]. Furthermore, the gut-associated lymphoid tissue (GALT) and immune cells present in the lamina propria located underneath the epithelial barrier have a unique role in initiating immune responses against pathogens that reach the intestinal lumen. In this regard, IEC recognize pathogen-associated molecular patterns (PAMP) such as viral RNA using pathogen recognition receptors (PRR) such as cytosolic retinoic acid-inducible gene-I (RIG-I), melanoma differentiation-associated gene-5 (MDA-5) or endosomal TLR3 [3,8,9,10,11,12]. The activation of these receptors subsequently leads to the transcription of pro-inflammatory genes. Downstream, the activation of PRR results in the secretion of pro-inflammatory cytokines such as IL-8, GROα, CXCL10, CCL20, CCL5 as well as type I and III IFNs, such as IFNβ and IFNλ [3,7,8,9,11,12,13]. These mediators are directly involved in viral defense by attracting and activating local innate (monocytes, neutrophils, eosinophils) and adaptive (helper and cytotoxic T-cells) immune cells since they promote the translocation of immune cells to the site of infection to support an adequate immune response against viral pathogens [8,11,12].

Dendritic cells (DC) play a crucial role in the induction of adaptive immune responses by presenting viral antigens and activating T- and B-cells [14,15]. The PRR expressed by DC are also able to recognize PAMP which trigger DC maturation leading to upregulated co-stimulatory molecule and CCR7 expression, allowing DC migration to lymph nodes where naïve T-cells are located [14,15,16]. In addition, DC process the viral antigens for their presentation to CD8^+^ or CD4^+^ T-cells in the lymph nodes resulting in the activation, cell division and differentiation of T-cell subsets [10,14,15]. The functions of DC in host defense against viral pathogens are not limited to antigen presentation but the cytokines secreted by DC also play a crucial role in the development of appropriate immune responses. Cytokines such as IL-12p70, IL-15 and type I IFNs are known to contribute to the development of Th1-type immunity and therefore might contribute to antiviral immune responses [11,14,17,18]. In spite of the well-described roles of CD8^+^ T-cells in viral clearance, CD4^+^ T-cells are also key immune players in the fight against viral infections [19]. These CD4^+^ helper T-cells (Th) contribute to viral clearance by promoting B-cell antibody production, improving CD8^+^ cytotoxic T-cell (Tc) function by supporting cytokine and chemokine secretion from DC, regulating inflammatory responses as well as by directly mediating in viral clearance [18,19].

Breastfeeding has been associated with lower risk of suffering intestinal and respiratory infections [20,21,22]. Moreover, the WHO identified breastfeeding as a protective factor against diarrheal diseases [23]. Recently it was shown that longer duration of exclusive breastfeeding was associated with a later detection of enteric viral pathogens in the stool of infants [24]. Human milk is known to contain many bioactive factors such as immunoglobulins. In particular, secretory IgA as well as IgG are known to provide immunity to breastfed infants [25]. Besides immunoglobulins, human milk oligosaccharides (HMOS) are one of the main components in human milk for which antiviral properties have been described due to their ability to bind pathogens and thus, preventing infection of epithelial cells. HMOS were also shown to promote barrier integrity and supporting the development of balanced immune responses as reviewed previously [26]. Moreover, previous studies showed that HMOS isolated from pooled human milk supported the maturation of human monocyte-derived DC (moDC) and promoted regulatory T-cell differentiation [27].

One of the most abundant HMOS in human milk, namely 2′-fucosyllactose (2′FL), was found to ameliorate the severity and incidence of RV-induced diarrhea possibly through the promotion of intestinal maturation and/or supporting neonatal immune response as shown in a neonatal rat model [28,29]. In addition, the duration of RV-induced diarrhea was shortened and increased immune cell populations were observed in piglets fed a formula containing 2′FL [30,31]. However, although some studies suggested increased RV infectivity with 2′FL [32], other studies observed a reduced infectivity in MA104 cells in vitro [33]. Furthermore, 2′FL was shown to promote anti-inflammatory and immunomodulatory properties in vitro [34,35], and in vivo a dietary intervention with 2′FL was shown to improve the vaccination immune response in an influenza-specific murine vaccination model associated with increased vaccine-specific IgG1 and IgG2a levels [36]. Therefore, 2′FL might be able to support the immune response against viral pathogens.

Polyinosinic-polycytidylic acid (pIC), a synthetic analog of dsRNA, is widely used to mimic viral infections. Stimulation of HT-29 IEC with pIC significantly induced IL-8 [37], CXCL10 [37,38] and CCL20 [39] secretion and upregulated IFNβ and TLR3 mRNA expression [40]. In addition, some studies investigated the activation of intracellular signaling pathways upon exposure to cationic lipid Lyovec™-complexed pIC. Lyovec™ binds pIC and facilitates internalization which results in intracellular RIG-I activation [41], more closely resembling RV infection of IEC through activation of cytosolic RIG-I and MDA-5 receptors [42]. Owing to the unique roles of IEC and regarding the key interactions with underlying immune cells in the fight against viral pathogens, we aimed to develop an in vitro model to study the role of IEC and moDC in coordinating the immune response upon a viral trigger. Therefore, IEC were stimulated with the TLR3 agonist pIC directly or with Lyovec™-complexed pIC (LV-pIC) to determine the additional effects of pIC internalization on cytokine secretion. In addition, to study the effect of viral-exposed IEC on innate and adaptive immune responses, LV-pIC-exposed IEC were co-cultured with moDC. Alternatively, moDC were directly exposed to LV-pIC in the absence of IEC. Then, the ability of these moDC to instruct naïve CD4^+^ Th-cells was studied. Furthermore, the capacity of 2′FL to modulate immune responses in these models was studied in order to shed some light on the effects of human milk oligosaccharide 2′FL in the immune development against a viral trigger.

## 2. Results

### 2.1. Poly I:C Stimulated IEC Secrete Viral Defense Related Cytokines

IEC were grown in 48 well-plates and exposed to 10 µg/mL naked pIC or LV-pIC to study if the mode of delivery of pIC had an effect on the cytokine secretion. In addition, to study the effects of 2′FL, IEC were pre-incubated for 24 h with 2′FL (0.5% *w/v*) after which pIC stimulation was applied, the supernatant was collected, and cytokine and chemokine secretion were studied (Figure S1).

**Figure 1:**
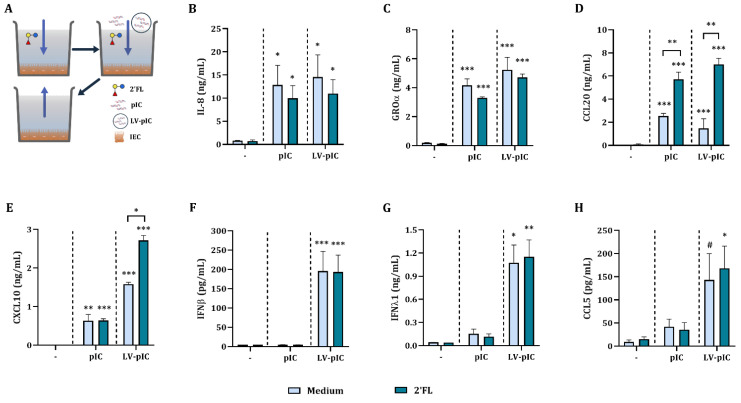
Cytokine and chemokine secretion from IEC upon stimulation with pIC. IEC were grown in 48-well plates until confluency and pre-incubated with 2′FL (0.5% *w*/*v*). After 24 h incubation, IEC were stimulated with naked or LV-pIC for 20 h after which the supernatant was collected (**A**) and IL-8 (**B**), GROα (**C**), CCL20 (**D**), CXCL10 (**E**), IFNβ (**F**) IFNλ1 (**G**) and CCL5 (**H**) were measured. Results are represented as mean ± SEM of *n* = 6 independent experiments, except for CXCL10 *n* = 4 (# *p* < 0.1, * *p* < 0.05, ** *p* < 0.01, *** *p* < 0.001).

Exposure to naked pIC significantly increased IL-8, GROα, CCL20 and CXCL10 concentrations as compared to medium control, but did not affect IFNβ, IFNλ1 and CCL5 (Figure 1). Pre-incubation with 2′FL did not have an effect on IL-8, GROα, CCL20, CXCL10, IFNβ, IFNλ1 and CCL5 concentrations, but significantly increased CCL20 concentrations of naked pIC stimulated IEC as compared to naked pIC stimulation alone (Figure 1C).

Exposure of IEC to pIC, either naked or LV-complexed, upregulated IL-8, GROα, CCL20, and CXCL10 concentrations (Figure 1B–E). Only upon exposure to LV-pIC IFNβ, and IFNλ1 were increased and CCL5 tended to increase (*p* = 0.06) (Figure 1F–H). Pre-incubation with 2′FL significantly increased CCL20 secretion of naked pIC-exposed IEC. Meanwhile, pre-incubation with 2′FL significantly increased CCL20 and CXCL10 secretion of IEC stimulated with LV-pIC (Figure 1D,E). The LV-pIC conditions were chosen for further studies due to the increased secretion in type I and III IFNs.

### 2.2. IEC Regulate the Cytokine Secretion from IEC/moDC Cultures Exposed to pIC

To study the regulatory function of IEC upon exposure to viral triggers, the cytokine secretion in IEC/moDC co-culture was assessed. In addition, the effect of 2′FL in IEC/moDC cultures was studied. Therefore, IEC were pre-incubated with 0.5% (*w*/*v*) 2′FL for 24 h, after which IEC were stimulated with 10 µg/mL LV-pIC in the presence of 2′FL. The stimulated IEC were then co-cultured with immature moDC for 48 h after which the secretion of cytokines and chemokines was measured in the basolateral supernatant. Alternatively, moDC were directly stimulated with LV-pIC in the presence of 2′FL (Figure S1).

**Figure 2:**
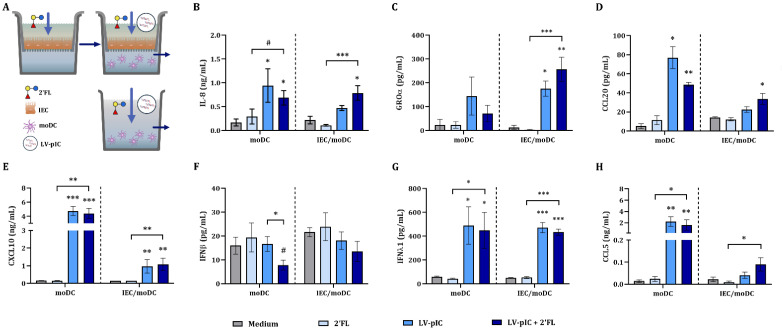
Cytokine and chemokine secretion upon stimulation with LV-pIC. IEC were grown in 12-well transwell inserts until confluency and pre-incubated with 2′FL (0.5% *w*/*v*). After 24 h pre-incubation, IEC were stimulated with LV-pIC and fresh 2′FL, and co-cultured with immature moDC for 48 h. Alternatively, moDC alone were stimulated with LV-pIC alone in the presence or absence of 2′FL for 48 h. After incubation, the basolateral supernatant was collected (**A**) and IL-8 (**B**), GROα (**C**), CCL20 (**D**), CXCL10 (**E**), IFNβ (**F**), IFNλ1 (**G**) and CCL5 (**H**) were measured. Results are represented as mean ± SEM of *n* = 6 independent moDC donors except for CCL20 *n* = 4 (# *p* < 0.1, * *p* < 0.05, ** *p* < 0.01, *** *p* < 0.001).

There was no effect in the cytokine secretion in moDC or IEC/moDC cultures exposed to 2′FL in absence of LV-pIC (Figure 2). Exposure of immature moDC to LV-pIC alone or in combination with 2′FL significantly increased IL-8, CCL20, CXCL10, IFNλ1 and CCL5 concentrations as compared to medium control and/or 2′FL alone (Figure 2B,D,E,G,H). LV-pIC did not affect GROα and IFNβ concentrations (Figure 2C,F). Exposure to 2′FL and LV-pIC did not further increase any of the cytokines and chemokines measured as compared to LV-pIC exposure alone, but reduced IFNβ (Figure 2F).

When immature moDC were co-cultured with IEC, exposure to LV-pIC alone or in combination with 2′FL significantly increased IL-8, GROα, CXCL10 and IFNλ1 concentrations as compared to medium control (Figure 2B,C,E,G). There was no effect on CCL20, IFNβ and CCL5 concentrations upon exposure to LV-pIC alone (Figure 2D,F,H). However, in combination with 2′FL, increased concentrations of CCL20 and CCL5 were observed as compared to medium control or 2′FL alone (Figure 2D,H).

LV-pIC exposure in the presence or absence of IEC resulted in differential effects regarding the release of mediators involved in viral defense. IL-8 and IFNλ1 were increased in IEC/moDC and moDC exposed to LV-pIC, GROα was only increased in the presence of IEC. CXCL10 was increased in IEC/moDC and moDC, but the concentrations remained relatively low in the presence of IEC. CCL20 and CCL5 secretion only increased in moDC cultures (in the absence of IEC), however 2′FL enhanced CCL20 and CCL5 secretion in IEC/moDC cultures.

### 2.3. Modulation of Galectin Secretion by LV-pIC and 2′FL

Galectins may play a role in viral defense and are known to be expressed by IEC and immune cells, therefore we studied the secretion of galectin-3, -4 and -9 in moDC alone or in IEC/moDC cultures (Figure S1).

**Figure 3:**
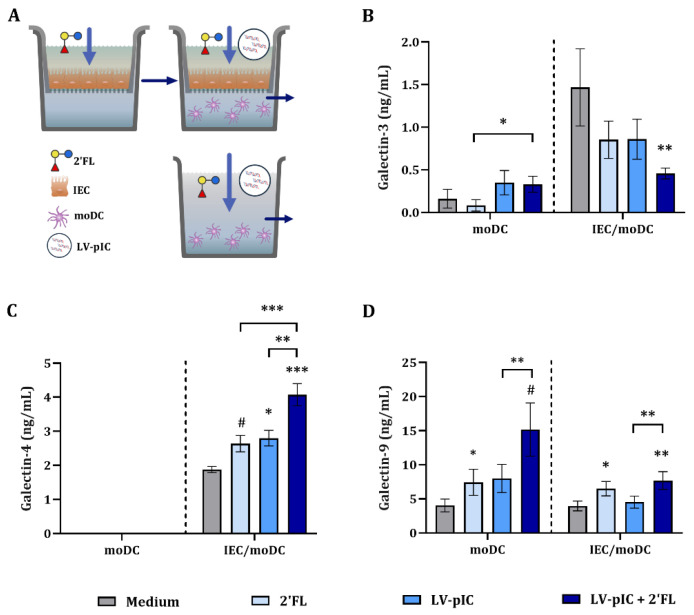
Galectin secretion upon stimulation with LV-pIC. IEC were grown in 12-well transwell inserts until confluency and pre-incubated with 2′FL (0.5% *w*/*v*). After 24 h pre-incubation, IEC were stimulated with LV-pIC and fresh 2′FL, and co-cultured with immature moDC for 48 h. Alternatively, moDC alone were stimulated with LV-pIC in the presence or absence of 2′FL for 48 h (**A**). After incubation, the basolateral supernatant was collected and galectin-3 (**B**), -4 (**C**) and -9 (**D**) were measured. Results are represented as mean ± SEM of *n* = 6 independent moDC donors (# *p* < 0.1, * *p* < 0.05, ** *p* < 0.01, *** *p* < 0.001).

There was no galectin-4 secretion by moDC alone (Figure 3C). Exposure to 2′FL or LV-pIC alone did not have an effect on galectin-3 but 2′FL significantly increased galectin-9 concentrations of moDC cultures (Figure 3B,D). However, combined exposure to LV-pIC and 2′FL significantly increased galectin-3 concentrations of moDC alone as compared to medium and/or 2′FL controls, while 2′FL significantly increased galectin-9 in LV-pIC-exposed moDC compared to medium control (Figure 3B,D).

In the IEC/moDC co-culture, exposure to 2′FL alone did not affect galectin-3 concentrations, but significantly increased galectin-9 and tended to increase galectin-4 concentrations (*p* = 0.08) as compared to medium control (Figure 3). Exposure to LV-pIC alone or in combination with 2′FL significantly decreased galectin-3 concentrations as compared to medium control and increased galectin-4 and -9 concentrations in the IEC/moDC co-culture (Figure 3).

IEC/moDC exposed to LV-pIC secreted increased galectin-4 concentrations. Combined exposure to LV-pIC and 2′FL further increased galectin-4, only in the presence of IEC, and galectin-9 in moDC cultures exposed to LV-pIC.

### 2.4. Dendritic Cell-Related Cytokine Secretion in IEC/moDC Model by LV-pIC

**Figure 4:**
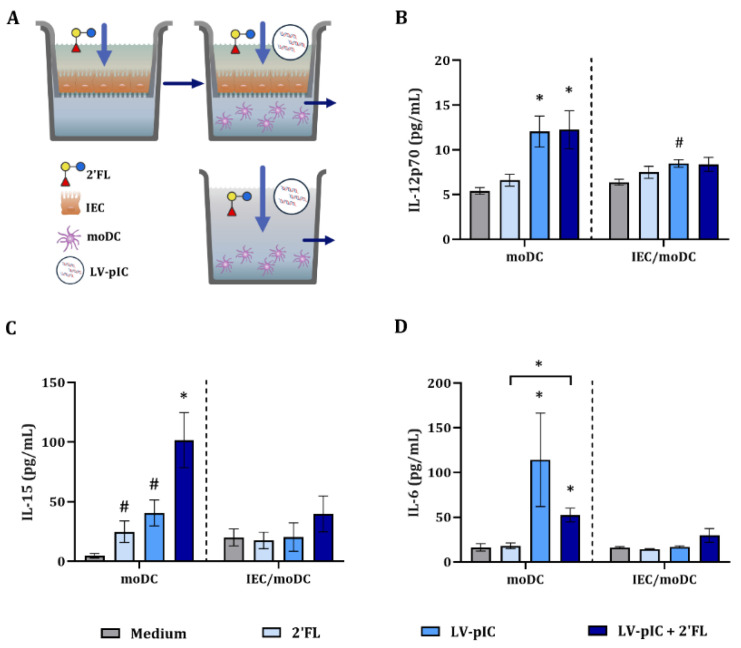
Cytokine secretion upon stimulation with LV-pIC. IEC were grown in 12-well transwell inserts until confluency and pre-incubated with 2′FL (0.5% *w*/*v*). After 24 h pre-incubation, IEC were stimulated with LV-pIC and fresh 2′FL, and co-cultured with immature moDC for 48 h. Alternatively, moDC alone were stimulated with LV-pIC in the presence or absence of 2′FL for 48 h (**A**). After incubation, the basolateral supernatant was collected and IL-12p70 (**B**), IL-15 (**C**) and IL-6 (**D**) concentrations were measured. Results are represented as mean ± SEM of *n* = 6 independent moDC donors (# *p* < 0.1, * *p* < 0.05).

To further study the effects of pIC on moDC, DC-derived pro-inflammatory cytokine secretion was studied in the presence or absence of IEC (Figure S1). There was no significant effect on the IL-12p70, IL-15 and IL-6 concentrations in moDC or IEC/moDC cultures upon incubation with 2′FL (Figure 4).

Significantly increased IL-12p70 and IL-6 concentrations were observed in LV-pIC-exposed moDC cultures in the presence or absence of 2′FL, but IL-15 was only increased in the presence of 2′FL and LV-pIC (Figure 4). 2′FL or LV-pIC alone did not affect IL-12p70, IL-15 and IL-6 concentrations (Figure 4) in the IEC/moDC cultures.

Increased pro-inflammatory IL-12p70, IL-15 and IL-6 secretion was observed in moDC cultures exposed to LV-pIC alone and/or in combination with 2′FL. This upregulation was not observed in IEC/moDC cultures.

### 2.5. Cytokine Secretion in DC/T-Cell Assay

In an allogeneic DC/T-cell assay we studied if ccDC (moDC derived from IEC/moDC or moDC cultures exposed to LV-pIC with or without 2′FL) were able to instruct T-cells. Therefore, ccDC were incubated with naïve T-cells for 5 days after which the cytokine secretion was studied (Figure S1).

**Figure 5:**
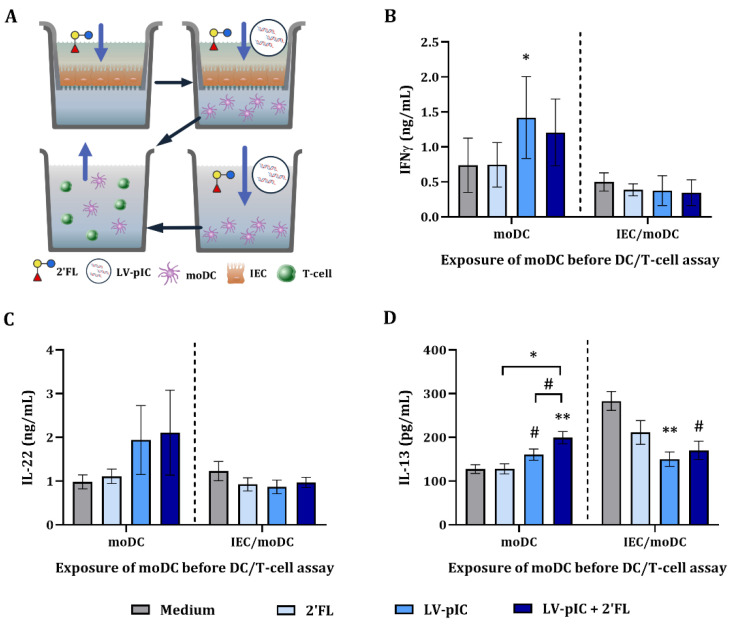
Cytokine secretion in DC/T-cell assay. Conditioned moDC (ccDC) previously exposed to LV-pIC activated IEC or to LV-complexed pIC directly, were incubated with naïve T-cells for 5 days (**A**) After incubation, the basolateral supernatant was collected and IFNγ (**B**), IL-22 (**C**) and IL-13 (**D**) were measured. Results are represented as mean ± SEM of *n* = 6 independent moDC donors (# *p* < 0.1, * *p* < 0.05, ** *p* < 0.01).

There was no effect on the IFNγ, IL-22 and IL-13 concentrations in the supernatant of DC/T-cells of ccDC that had been exposed to 2′FL (Figure 5). ccDC from LV-pIC-exposed cultures significantly increased IFNγ and tended to increase IL-13 (*p* = 0.08) concentrations in DC/T-cell cultures, as compared to medium control. Increased IL-13 secretion was observed in DC/T-cell cultures from LV-pIC and 2′FL exposed to moDC cultures as compared to medium control (Figure 5D). Meanwhile, IL-22 concentrations remained unchanged (Figure 5C).

No effect was observed on IFNγ and IL-22 concentrations from DC/T-cell supernatants of ccDC derived from IEC/moDC cultures incubated with 2′FL, (Figure 5B,C). However, in DC/T-cell supernatants from ccDC derived from IEC/moDC cultures exposed to LV-pIC alone or in combination with 2′FL, significantly decreased IL-13 concentrations were observed (Figure 5D). Upon combined exposure of LV-pIC and 2′FL, IL-13 concentrations tended to decrease (*p* = 0.08) (Figure 5D). IL-10, IL-17A and IL-23 concentrations remained under the detection limit. Additionally, the phenotype of the T-cells was studied after DC/T-cell assay. There was no effect on the percentages of CD4^+^, CD69^+^ or CD69^+^CXCR3^+^ cell populations (Figure S2).

DC/T-cell cultures from moDC exposed to LV-pIC increased Th1-type IFNγ and Th2-type IL-13. Meanwhile, in DC/T-cell cultures from ccDC derived from IEC/moDC conditions, IFNγ secretion was not affected, and decreased IL-13 concentrations were observed.

## 3. Discussion

Besides providing a barrier, IEC contribute to host defense by regulating PRR expression and crosstalk with underlying immune cells, as well as regulating cytokine and chemokine secretion [4,11]. Here, we study the involvement of IEC in the development of immune responses against a viral trigger and the modulation by 2′FL, an HMOS abundant in human milk.

To set up an enteric viral infection model, a synthetic analog of dsRNA, namely pIC, was used. Therefore, pIC was added directly on top of the IEC monolayer (naked pIC) and compared to LV-complexed pIC (LV-pIC), which supports the passage of pIC through the cellular membrane into the cytosol [43]. Widely used to mimic a viral trigger, pIC is known to activate TLR3 and was found to promote a significant increase in IL-8, CXCL10 and CCL20 secretion from stimulated IEC cells [37,38,39]. These results are in line with our observations as shown in this manuscript, where upregulated IL-8, CXCL10 and CCL20 release was observed upon either naked or LV-pIC stimulation. Furthermore, pIC stimulated IEC also secreted significantly increased GROα levels independent of LV-complexation, which to our knowledge has not been previously shown. However, in HT-29 cells infected with an enteric virus such as RV, upregulated IL-8 and GROα concentrations were observed [44], suggesting that stimulation of IEC with either naked or LV-pIC might mimic to some extent an enteric viral infection similar to RV.

In addition, upon infection of IEC with RV, increased IFNβ secretion was observed which was suggested to be mediated by RIG-I and MDA-5 signaling [12,41,42,45]. Besides TLR3, pIC was shown to activate RIG-I and MDA-5 [46]. Here, IFNβ was not detectable upon exposure to naked pIC. Only exposure to LV-pIC, promoted IFNβ secretion from IEC cells, suggesting that naked pIC might not be able to fully stimulate cytosolic RIG-I and MDA-5 signaling, and thus failed to promote IFNβ secretion, as opposed to LV-pIC. These results suggest that the mode of delivery of pIC results in the activation of different signaling pathways and that only upon LV-complexation of pIC a strong activation of RIG-I and MDA-5 is obtained, leading to IFNβ secretion in HT-29 cells.

Not only type I IFNβ secretion was increased by LV-pIC. An upregulation of type III IFNλ1 and a tendency towards increased CCL5 secretion was also observed upon stimulation of IEC with LV-pIC. Both type I IFNβ and type III IFNλ1 secretion by infected cells is known to signal in an autocrine and paracrine manner to surrounding cells which, by activating IFN-stimulated genes (ISG), induce a local antiviral state [11,12,13]. Similarly, this autocrine and paracrine activation loop might result in further stimulation of CCL5 secretion by amplification of IFN production and induction of the activation of other ISG [47]. Type I IFNs are key factors contributing to the modulation of antiviral immune responses by suppressing viral replication, while type III IFNs are particularly important for innate immune responses at mucosal barriers such as the gut barrier [48,49]. Interestingly, previous studies observed increased CCL5 secretion in RV-infected HT-29 cells [44]. These results support the idea that LV-pIC stimulation of IEC might mimic to some extent the immune responses observed upon an enteric viral infection.

Dendritic cells act as a bridge between the innate and adaptive immune responses and as such, their interaction and crosstalk with IEC is key to maintaining immune homeostasis in the fight against pathogens such as viruses [4,7,11]. In this regard, the contribution of IEC in supporting moDC activity in response to a viral trigger was studied. Upon stimulation of moDC with LV-pIC, significantly increased CCL20, CXCL10 and CCL5 secretion was observed, as opposed to IEC/moDC cultures where secretion of such chemokines was limited. On the contrary, GROα was only increased in IEC/moDC cultures while IL-8, IFNβ and IFNλ1 were induced equally in IEC/moDC and moDC cultures. GROα is known to function in an autocrine regulatory manner leading to the promotion of cell survival and immunomodulation in IEC [50] which might have led to its increased secretion in IEC/moDC cultures. In line with our results, an improved antiviral state was observed by increased CXCL10 and IFNβ as well as decreased IL-8 in a co-culture model using porcine intestinal cells and immune cells upon exposure to probiotics [8,51]. Our results indicate that IEC might selectively regulate CCL20, CXCL10 and CCL5 in order to restrict the propagation of the viral infection and the subsequent tissue damage, thus promoting local clearance of the virus and facilitating re-establishment of tissue homeostasis [4,52]. However, when the virus crosses the epithelial lining and DC are fully exposed to the virus without the regulatory action of epithelial cells, a much stronger immune activation is generated with the induction of systemic adaptive immune response to the viral trigger.

Due to the benefits of breastfeeding in the protection against infections [20,21,22], we studied the ability of HMOS in modulating the cytokine and chemokine secretion in IEC and IEC/moDC cultures exposed to LV-pIC. In particular, one of the most abundant HMOS, namely 2′FL, showed anti-inflammatory and immunomodulatory properties in vitro [34,35] as well as a reduction in the incidence and severity of RV-induced diarrhea in vivo [28,29,30,31]. Here, we observed that pre-incubation of IEC with 2′FL and subsequent stimulation with pIC significantly increased CCL20 and/or CXCL10 concentrations compared to activation with pIC alone. Moreover, in the IEC/moDC or moDC cultures, no effect was observed upon 2′FL exposure, except for a slight increase in CCL20 and CCL5 secretion in IEC/moDC cultures when combined with LV-pIC. These chemokines are known to be involved in DC, monocyte and T-cell (Th and Tc) recruitment or NK cell activation and might be beneficial to induce a proper anti-viral state [8,11,12,53,54,55,56] since these can promote an appropriate immune response against the encountered threat. Furthermore, 2′FL was found to selectively enhance the secretion of chemokines in LV-pIC-exposed IEC or IEC/moDC cultures suggesting possible immunomodulatory roles, which may be beneficial in viral defense. Further studies are needed to confirm the ability of 2′FL to enhance mucosal defense and protect against specific enteric viruses using viral proteins and/or inactivated viruses.

Beyond the cytokine and chemokine functions on the immune response against viruses, galectins have also shown to be key regulators of many immune processes. In the current study, galectin-4 and -9 secretion was further increased by 2′FL in LV-pIC stimulated IEC/moDC cultures, while in moDC cultures galectin-9 and -3 were increased. These results point towards the involvement of galectins in the regulation of immune responses against pIC and the ability of 2′FL in modulating galectin secretion in response to a viral trigger. Galectins were shown to bind viral glycans and PRR extracellularly and interact with viral and cytosolic components present in the cytoplasm and therefore, might be able to modulate immune responses [57,58,59]. Furthermore, increased galectin levels, and in particular galectin-9 levels, were observed upon viral infections [60] to dengue [61], influenza [62], HIV [63], hepatitis B and C [64,65] as well as COVID-19 [66] as compared to healthy controls. Furthermore, recent studies have suggested the use of plasma concentrations of galectins as biomarkers for disease prognosis since elevated plasma galectins were linked to higher viral load or more severe infection. Galectins are probably produced as protective factors with regulatory functions that can boost immunity and thereby, promote host defense [61,67,68,69].

Although the exact mechanism is still unknown, it is hypothesized that galectins might promote the induction of cytokines and immune cells to support viral immune defense. In particular galectin-9 was shown to act as damage associated molecular patterns (DAMP) and to induce immunomodulation of various immune cells [70]. However, galectin-9 plasma levels also correlated with pro-inflammatory mediator secretion (IL-6, TNFα, CXCL10) in dengue and COVID-19 infected patients [69,71]. In addition, galectins are known to exert biological functions that could contribute to host defense such as cell adhesion and migration [71], Treg cell differentiation and function [72], suppressing CD4^+^ Th and CD8^+^ Tc [73] or controlling apoptosis to reduce tissue damage [73,74,75] as well as affecting DC maturation [76]. Furthermore, circulating galectins were shown to be associated with enhanced vaccine-specific immune responses in a murine influenza vaccination model [77]. Galectins are associated with various immune processes and as such, can interact with many innate immune cells to support host defense against viruses. Innate immune cells are able to secrete galectins which can result in the modulation of innate and adaptive immune cells at the site of infection. Here they may regulate immune responses and thereby contribute to the resolution of the viral infection, as well as keeping local tissue homeostasis by regulating and limiting exaggerated immune responses which could lead to tissue damage.

The secretion of pro-inflammatory cytokines IL-12p70 and IL-6 was induced in moDC exposed to LV-pIC, and also IL-15 was secreted but only in the presence of LV-pIC and 2′FL, as opposed to IEC/moDC cultures. This supports the regulatory role of IEC in promoting local viral clearance and tissue homeostasis while downregulating excessive immune activation. Both IL-6 and IL-12 are produced upon DC activation and contribute to DC/T-cell communication and immune cell recruitment to the site of infection or inflammation [78]. Meanwhile IL-15 is known to induce DC differentiation and thereby promoting Th1-type immune responses in the intestine [17]. Particularly, IL-12p70 secretion and increased DC maturation were observed in immature DC stimulated with TLR3 ligand pIC as shown by an increase in the expression of CD80, CD86 and MHC-II markers [79]. In activated T-cells, IL-15 promoted IFN**γ** production as well as synergizing with IL-12 to upregulate IFN**γ** production [80]. Meanwhile, the differentiation of naïve CD4^+^ Th-cells and the production of CD8^+^ Tc-cells were induced by IL-6 and IL-15, respectively [17,80,81,82,83]. Furthermore, IL-6 and IL-15 are known to contribute to tissue protection [17,81]. In this manuscript, we observed that when DC were co-cultured with naïve T-cells, these LV-pIC-exposed moDC, were capable of activating CD4^+^ Th-cells as shown by increased Th1-type IFN**γ** and Th2-type IL-13 secretion in the presence of 2′FL. DC producing high amounts of IL-12p70 and IL-15 are known to drive Th1-type immune responses [14,17,80,82]. Therefore, we suggest that the increased IFN**γ** secretion observed in DC/T-cell cultures from LV-pIC-stimulated moDC might be associated with the Th1-promoting cytokine secretion seen in moDC cultures. Contrarily, no increase in T-cell activation was observed in DC/T-cell cultures from IEC/moDC conditions, indicating a possible regulatory role of IEC in suppressing the instruction of DC towards promoting a general systemic inflammatory response.

Overall, LV-pIC was found to induce a full repertoire of mediator release by IEC which are involved in viral defense, of which CCL20 and CXCL10 were further enhanced by 2′FL. LV-pIC exposure to moDC also induced inflammatory mediator release associated with Th1 cell activation, as opposed to IEC/moDC cultures. In the latter, 2′FL was found to enhance galectin-4 and -9 secretions during LV-pIC exposure, which may help to control local immune activation and maintain tissue homeostasis during viral defense. In this novel in vitro mucosal viral defense model using viral RNA, we observed a unique role of IEC in regulating immune responses against a viral trigger. This suggests a tight control of local intestinal viral defense which may be supported by dietary components such as 2′FL.

## 4. Materials and Methods

### 4.1. Intestinal Epithelial Cell Culture

Human HT-29 cell line (ATCC, HTB-38, Manassas, VA, USA) was used as IEC. The cells were cultured in 75 cm^2^ flasks (Greiner Bio-One, Alphen aan den Rijn, The Netherlands) using Mc Coy 5A medium (Gibco, Invitrogen, Carlsbad, CA, USA) supplemented with 10% fetal calf serum (FCS), penicillin (100 U/mL) and streptomycin (100 µg/mL) (both from Sigma-Aldrich, St. Louis, MO, USA). IEC were kept in incubation at 37 °C and 5% CO_2_ and medium was refreshed every 2–3 days.

### 4.2. IEC Model

IEC were seeded (50.000 cells/well) in 48-well plates (Costar Corning Incorporated, New York, NY, USA) and grown until confluency in Mc Coy 5A medium. Medium was refreshed every 2–3 days. When IEC reached confluency, cells were pre-incubated with 0.5% (*w*/*v*; 5 mg/mL) 2′FL solutions (>90% pure, produced by microbial fermentation) for 24 h. After the pre-incubation period, medium was removed and IEC were stimulated with 10 µg/mL high-molecular weight pIC (Invivogen, San Diego, CA, USA) either naked or complexed with Lyovec™ (LV) (Invivogen), alone or in the presence of 2′FL, for 20 h after which, the supernatant was collected and stored for cytokine analysis.

### 4.3. Peripheral Blood Mononuclear Cell Isolation

Buffy coats from healthy donors (who had given informed consent) were used to isolate human peripheral blood mononuclear cells (PBMC) (Blood bank, Amsterdam, The Netherlands) by density gradient centrifugation using pre-filled Leucosep™ tubes (1000× *g*, 13 min, Greiner Bio-One). The isolated lymphocyte fraction was washed with PBS supplemented with 2% FCS and the remaining erythrocytes were lysed using a red blood cell lysis buffer (4.14 g NH_4_Cl, 0.5 g KHCO_3_, 18.6 mg Na_2_EDTA in 500 mL demi water, sterile filtered, pH = 7.4). The isolated PBMC fraction was resuspended in RPMI 1640 supplemented with 10% FCS, penicillin (100 U/mL) and streptomycin (100 µg/mL).

### 4.4. Monocyte Isolation and Culture

A negative selection MACS kit was used to isolate CD14^+^ cells from PBMC following manufacturer’s protocol (Miltenyi Biotec, Bergisch Gladbach, Germany). RPMI 1640 supplemented with 10% FCS, IL-4 (100 ng/mL), GM-CSF (60 ng/mL, both from Prospec Rehovot, Israel) penicillin (100 U/mL) and streptomycin (100 µg/mL) was used to culture isolated CD14^+^ cells for 7 days. Medium was refreshed on days 3 and 6 of culture. On day 7, immature moDC were collected.

### 4.5. IEC/moDC Co-Culture Model

One week before the experiments, IEC were seeded in 12-well transwell inserts diluted 8–10 times based on surface area (Corning). Medium was changed every 2–3 days (37 °C, 5% CO_2_). When confluency was achieved, IEC were pre-incubated with 0.5% 2′FL (*w*/*v*) for 24 h. After the pre-incubation, medium was removed and IEC/moDC co-culture was started. Therefore, immature moDC (0.5 × 10^6^ cell) were cultured in the presence or absence of IEC. Apically, 10 µg/mL Lyovec™complexed pIC was added and 2′FL solution was refreshed and incubated for 48 h after which supernatant was collected. Additionally, conditioned moDC (ccDC) were collected and co-cultured with naïve T-cells.

### 4.6. DC/T-Cell Assay

A negative selection MACS kit was used to isolate CD4^+^CD45RA^+^ naïve T-cells from PBMC, following manufacturer’s protocol (Miltenyi Biotec). IMDM medium supplemented with 10% FCS, 20 µg/mL apotransferrine (Sigma), β-mercaptoethanol (Sigma), penicillin (100 U/mL) and streptomycin (100 µg/mL) was used to co-culture isolated naïve T-cells (1 × 10^6^ cell/well) with ccDC (0.1 × 10^6^ cell/well) in 24 well flat-bottomed plates for 5 days. After incubation, supernatant was collected and stored for further analysis. Additionally, cells from DC/T-cell cultures were stained for flow cytometry analysis.

### 4.7. Enzyme-Linked Immunosorbent Assay (ELISA)

Supernatants from IEC, IEC/moDC and DC/T-cell assays were analyzed for cytokine and mediator secretion. Commercially available kits were used to determine IL-8, CXCL10, CCL5, GROα, IFNλ1, IFNβ, IL-22, galectin-3 (from R&D systems, Minneapolis, MN, USA), IFNγ, IL-13, (from Thermo Fischer scientific, Waltham, MA, USA), secretion according to manufacturer’s protocol. Human galectin-4 and -9 were measured using antibody pairs (R&D systems). In short, high-binding Costar 9018 plates were incubated overnight at 4 °C with 0.75 µg/mL human galectin-4 or -9 affinity purified polyclonal antibody. Non-specific binding was blocked with 1% bovine serum albumin (BSA, Roche Diagnostics, Mannheim, Germany) in PBS for 1 h after which plates were washed and streptavidin-HRP (R&D systems) was added and incubated for 40 min. After washing, tetramethylbenzidine was used as a substrate to develop the reaction (TMB, Thermo Fischer scientific), which was stopped with 1 M H_2_SO_4_. Optical density was measured at 450 nm.

### 4.8. Flow Cytometry

After DC/T-cell assay immune cells were stained for flow cytometry analysis. Cells from DC/T-cell cultures were incubated with Fc receptor blocking solution (Biolegend, San Diego, CA, USA) for 10 min on ice. Then, cells were washed in PBS supplemented with 1% BSA and incubated for 30 min on ice with the following antibodies: CD4-PerCp Cy5.5, CD69-eFluor 450 and CXCR3-Alexa Fluor 488 (all from Thermofisher except CXCR3 from BD). Dead cells were excluded using Fixable Viability Dye eFluor^®^ 780 (Thermofisher). Stained cells were measured by FACS Canto II (BD Biosciences) and analyzed using Flowlogic software version 7 (Inivai Technologies, Mentone, VIC, Australia).

### 4.9. Statistical Analysis

All statistical analysis were performed using GraphPad Prism software version 8 (San Diego, CA, USA). Data were transformed prior to ANOVA analysis if they did not fit normal distribution and/or homogeneity of variance. One-way repeated measures ANOVA followed by Bonferroni’s post hoc test with selected pairs were used for statistical analysis. The Geisser–Greenhouse correction was applied if the criteria for homogeneity of variance were not met. Non-parametric Friedman test with Dunn’s post hoc was conducted in case the data did not meet normal distribution after transformation. The conditions with naked pIC and LV-pIC as well as moDC cultures and IEC/moDC cultures were analyzed separately as represented by the dotted line. Probability values of *p* < 0.05 were considered significant.

## Data Availability

The data presented in this study are available upon request from the corresponding author.

## Supplementary Materials

The following supporting information can be downloaded at: https://www.mdpi.com/article/10.3390/ijms231810958/s1.
