# Supplementary material for: Human Milk Oligosaccharide 2′-Fucosyllactose Modulates Local Viral Immune Defense by Supporting the Regulatory Functions of Intestinal Epithelial and Immune Cells
Source: Int J Mol Sci. 2022 Sep 19;23(18):10958. doi: 10.3390/ijms231810958 (PMC9506168; doi:10.3390/ijms231810958)
Supplement: Supplementary file 1 [file ijms-23-10958-s001.zip › ijms-1892718-supplementary.pdf]

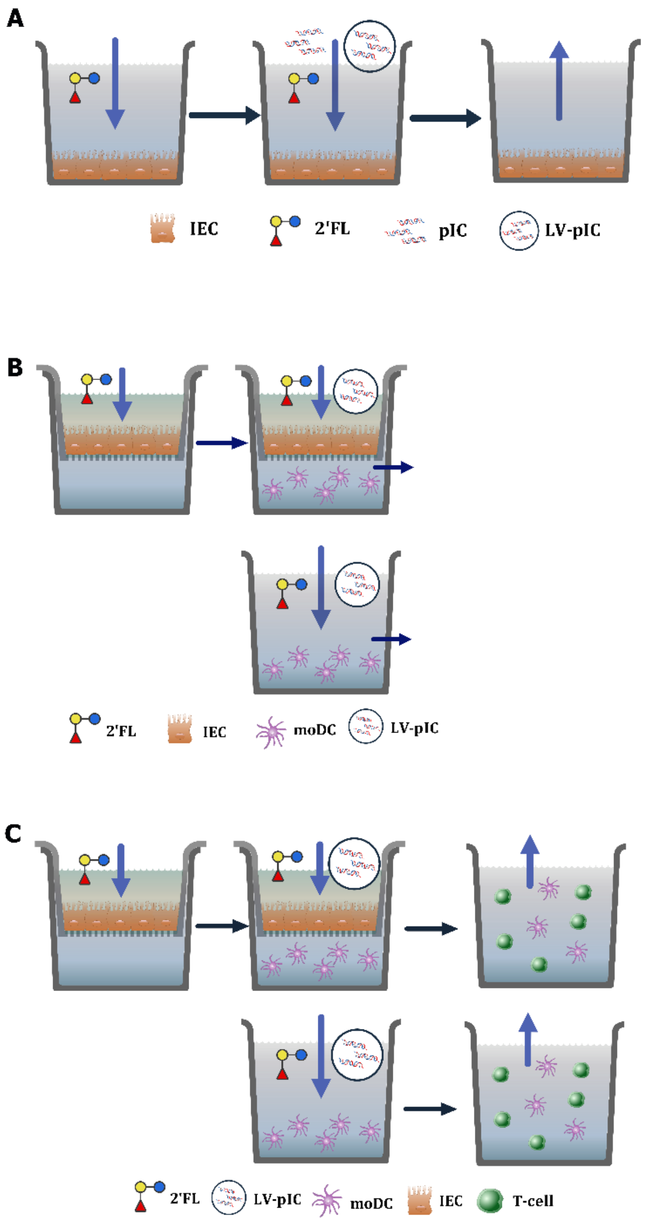

Figure S1. Overview of the experimental design. IEC cells form the first line of defense against viral infections and alarm underlying innate immune cells via inflammatory mediator release. HMOS may be able to modify this response. To set up a model to study mucosal viral infection using pIC as a viral mimic, we first compared the effect of the mode of delivery of pIC on the cytokine and chemokine secretion by IEC. Therefore, naked pIC was compared to Lyovec™-complexed (LV-pIC). LV was used to facilitate pIC internalization. In addition, the immunomodulatory capacity of HMOS 2′FL to regulate epithelial activation upon naked pIC or LV-pIC exposure was studied (**A**). The ability of the activated IEC to regulate the sequential immune response after exposure to the viral trigger was also studied. Therefore, moDC were cultured in the presence or absence of IEC and exposed to LV-pIC alone or in combination with 2′FL (**B**). To study the functional outcome of moDC maturation after LV-pIC exposure in the presence or absence of IEC the capacity of these conditioned DC to instruct T-cells responses was studied. Therefore after the IEC/moDC or moDC cultures, moDC were harvested and subsequently co-cultured with naïve T-cells after which the T-cell cytokine release was determined as a reflection of generation of an adaptive immune response upon a viral trigger (**C**).

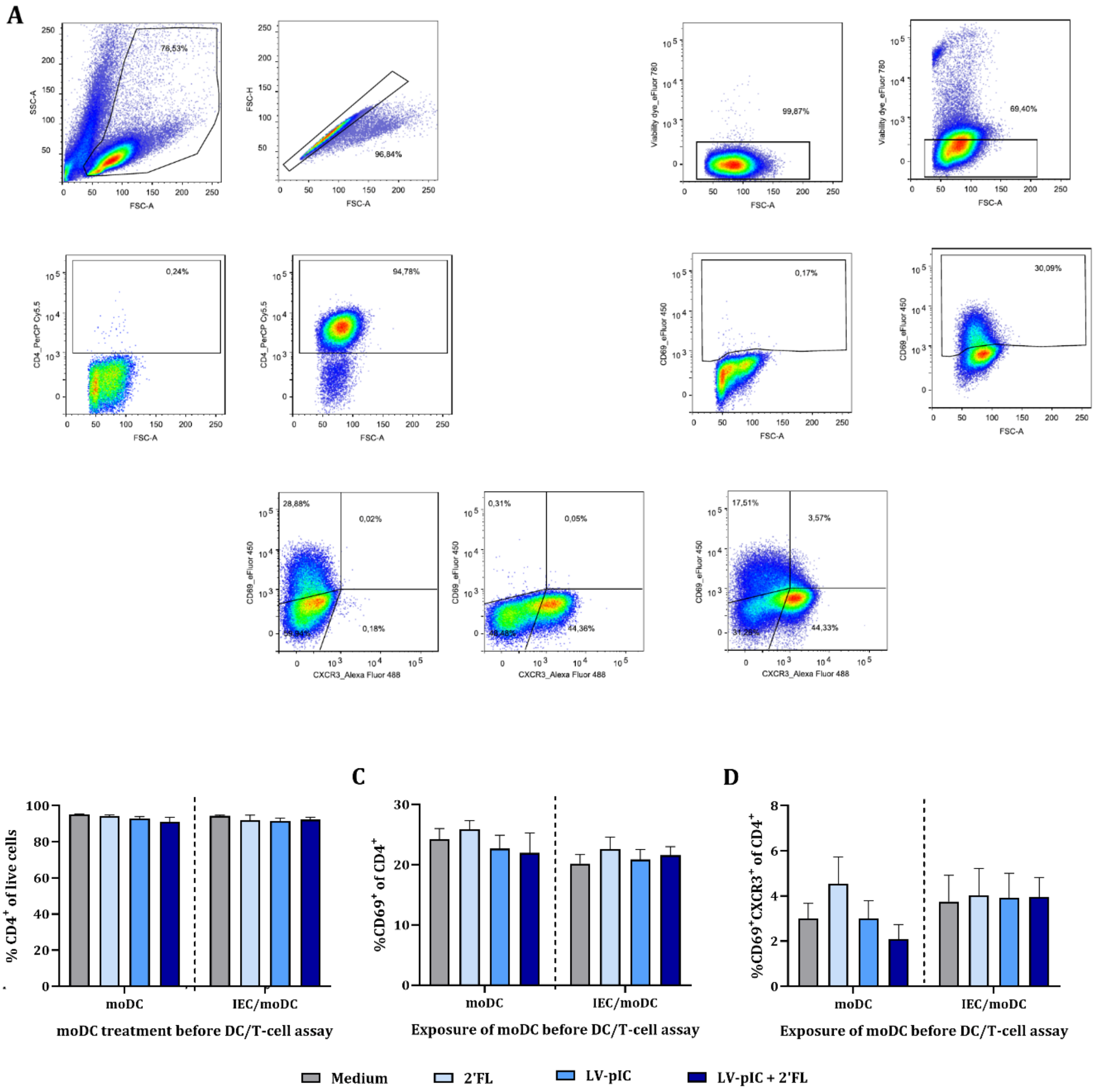

Figure S2. Phenotype of T-cells after DC/T-cell assay. Conditioned moDC previously exposed to LV-pIC conditioned IEC or to LV-complexed pIC directly, were incubated with naïve T-cells for 5 days after which the phenotype was studied. Representative plots showing the used gating strategy are shown (**A**). The percentage of CD4^{+} (**B**), CD69^{+} (**C**) and CD69^{+}CXCR3^{+} (**D**) are shown. Data are represented as mean ± SEM of n = 6 independent moDC donors.
